# A Case of Erysipelas

**Published:** 1888-05

**Authors:** H. P. Holmes

**Affiliations:** Sycamore, Ill.


### A CASE OF ERYSIPELAS.

H. P. Holmes, M.D., Sycamore, Ill.

I was called, February 17th, to see Mrs. V——, aged fifty-seven years. Found her sick in bed, very weak, and slightly delirious. Her face was deeply flushed and swollen. The eyelids were swollen so that it was impossible to open them, the swelling here being of a light color and consequently œdematous. The patient was quite restless. There was no thirst; the pulse was 128 and the temperature 104.45. The urine was greatly diminished in quantity and of a dark, brownish color, loaded with earthy phosphates.

The diagnosis was erysipelas and the prognosis, considering the temperature, age of the patient, and delirium, very grave. The family had been using upon the face a wash composed of Quassia, Sal Soda, Sugar of Lead, Saltpeter, Amm. carb., and Opium, and were very reluctant to give up its use, as it had been known to cure (?) many patients when nothing else would. I had had some experience with the application not the most satisfactory to my patient, although it had rapidly removed the erysipelatous blush, and I refused to treat this patient if the wash was to be used. The case was put into my hands and received a few pellets of Apis mel.$^{200}$ in half a glass of water, a teaspoonful every hour until improvement was noticed; then take every two hours.

The next day the patient was very much better in every way. Pulse 95 and temperature 101. The swelling had gone down fully half and the eyes could be opened easily. Put the patient upon placebo and asked for a report next day. That evening patient reported more restlessness and a burning sensation in the face, but the general appearance improving. Arsenicum$^{200}$ was prescribed as before. This prescription, made on the 19th, was all the medicine the patient needed to complete the cure.

February 22d I was again called, in the night, to prescribe for the patient, as her sister feared an attack of apoplexy. Her face was much flushed, her breathing heavy, and difficult to rouse her from her sleep. Her father having died from an apoplectic stroke, it was the cause of alarm in this case. There was apparently no farther trouble from the erysipelas. I sent a single powder of Belladonna$^{200}$ which removed the trouble *cito tuto et jocunde.* I saw the patient the next day, and every symptom of the erysipelas had disappeared. I then gave a single dose of Sulphur$^{200}$ to finish the case, and had the satisfaction of seeing a case recover that under average practice would very likely have ended in death.

The points worthy of remark in this case are as follows: Erysipelas is not a *local* disease, many authorities and general practice to the contrary notwithstanding. Local treatment is not certain, and is, as a rule, productive of bad results. To remove the erysipelatous hue by local treatment is not to cure the disease, and such suppressive treatment will most certainly be followed by some bad result. On the other hand, systematic treatment, which leaves the blush alone that it may stand as a landmark and indicate the progress of the cure, is the only certain and satisfactory treatment. Erysipelas being one of our worst diseases to treat, a cure effected with 200ths alone shows that it is not necessary to increase the dosage in proportion as the disease is considered dangerous. Furthermore, the repetition of the dose, in the way the above case was treated, is not only not dangerous, but with me efficacious as a rule. While a single dose of the indicated remedy might have cured the case as well, if not better, than the same sized dose divided as above, I did not, in this case, care to leave the patient upon a placebo after a single dose of the indicated remedy. In this case the remedy was not repeated at the next visit, but was left to act until improvement ceased. The giving of a dose of Sulphur high to finish up a case is a style of practice that has been very successful with me. I do not intend to say that it is prescribed as the *similia*, but the practice is followed by many good physicians, and is usually successful. I think the reason is that there are very few patients but what a dose of Sulphur at any time will benefit them.

